# Mutations in *trpγ*, the homologue of *TRPC6* autism candidate gene, causes autism-like behavioral deficits in *Drosophila*

**DOI:** 10.1038/s41380-022-01555-1

**Published:** 2022-05-02

**Authors:** Angelina Palacios-Muñoz, Danielle de Paula Moreira, Valeria Silva, Isaac E. García, Francisco Aboitiz, Mehdi Zarrei, Gabriele Campos, Olivia Rennie, Jennifer L. Howe, Evdokia Anagnostou, Patricia Ambrozewic, Stephen W. Scherer, Maria Rita Passos-Bueno, John Ewer

**Affiliations:** 1 Centro Interdisciplinario de Neurociencia de Valparaíso, Universidad de Valparaíso, Valparaíso, Chile; 2 Laboratorio de Genética y Conducta, Facultad de Odontología, Universidad de Valparaíso, Valparaíso, Chile; 3 Centro de Investigación Interoperativo en Ciencias Odontológicas y Médicas, Facultad de Odontología, Universidad de Valparaíso, Valparaíso, Chile; 4 Departamento de Psiquiatría, Escuela de Medicina, y Centro Interdisciplinario de Neurociencias, Pontificia Universidad Católica de Chile, Santiago, Chile; 5 Centro de Estudos do Genoma Humano e Células Tronco, Departamento de Genética e Biologia Evolutiva, Instituto de Biociências, Universidade de Sao Paulo, São Paulo, Brasil; 6 Laboratorio de Fisiología Molecular y Biofísica, Facultad de Odontología, Universidad de Valparaíso, Valparaíso, Chile; 7 The Centre for Applied Genomics, The Hospital for Sick Children, Toronto, ON Canada; 8 Program in Genetics and Genome Biology, The Hospital for Sick Children, Toronto, ON Canada; 9 Holland Bloorview Kids Rehabilitation Hospital, University of Toronto, Toronto, ON Canada; 10 Autism Research Unit, Developmental Pediatrics, The Hospital for Sick Children, Toronto, ON Canada; 11 Department of Molecular Genetics and McLaughlin Centre, University of Toronto, Toronto, ON Canada; 12 Instituto de Neurociencia, Facultad de Ciencias, Universidad de Valparaíso, Valparaíso, Chile

**Keywords:** Neuroscience, Autism spectrum disorders, Genetics

## Abstract

Autism Spectrum Disorder (ASD) is characterized by impaired social communication, restricted interests, and repetitive and stereotyped behaviors. The *TRPC6* (*transient receptor potential channel 6*) represents an ASD candidate gene under an oligogenic/multifactorial model based on the initial description and cellular characterization of an individual with ASD bearing a de novo heterozygous mutation disrupting *TRPC6*, together with the enrichment of disruptive *TRPC6* variants in ASD cases as compared to controls. Here, we perform a clinical re-evaluation of the initial non-verbal patient, and also present eight newly reported individuals ascertained for ASD and bearing predicted loss-of-function mutations in *TRPC6*. In order to understand the consequences of mutations in *TRPC6* on nervous system function, we used the fruit fly, *Drosophila melanogaster*, to show that null mutations in *transient receptor gamma* (*trpγ*; the fly gene most similar to *TRPC6*), cause a number of behavioral defects that mirror features seen in ASD patients, including deficits in social interactions (based on courtship behavior), impaired sleep homeostasis (without affecting the circadian control of sleep), hyperactivity in both young and old flies, and defects in learning and memory. Some defects, most notably in sleep, differed in severity between males and females and became normal with age. Interestingly, hyperforin, a *TRPC6* agonist and the primary active component of the St. John’s wort antidepressant, attenuated many of the deficits expressed by *trpγ* mutant flies. In summary, our results provide further evidence that the *TRPC6* gene is a risk factor for ASD. In addition, they show that the behavioral defects caused by mutations in *TRPC6* can be modeled in *Drosophila*, thereby establishing a paradigm to examine the impact of mutations in other candidate genes.

## Introduction

Autism Spectrum Disorder (ASD, or autism) is a neurogenetic developmental disorder diagnosed solely on the basis of behavior, characterized by hallmark features including impaired social communication, restricted interests, and repetitive and stereotyped behaviors [1]. There is great phenotypic heterogeneity among ASD patients and symptoms often co-occur with other psychiatric and neurological conditions (e.g., intellectual disability, ID > 40%; attention-deficit hyperactivity disorder, ADHD > 50%; sleep, and anxiety disorders [2–6]) as well as with atypical sensory processing and integration (>90%), which affects every sensory modality [7]. ASD affects ~1% of individuals worldwide, exhibiting a 4:1 male to female sex bias [8, 9], a bias that is also observed in ADHD, a disorder that can share a genetic bases with ASD [10–13].

Genomic analyses have contributed greatly to our understanding of the genetic architecture of ASD, and led to the identification of some highly-penetrant bona fide ASD-relevant genes [14] as well as of hundreds of other risk genes [15–17]. Disruption of these ASD-relevant genes by rare pathogenic variants (single nucleotide variants, indels, or copy number variations or CNVs) are responsible for most monogenic forms of the disorder [16, 18–22]. However, much work has yet to be done to understand the genetic basis of oligogenic/multifactorial inheritance of ASD, which represents the majority of cases [23–25]. In addition, a major remaining challenge is determining how mutations in particular neurodevelopmental genes trigger specific core behavioral symptoms and, in addition, why certain comorbidities arise. In this regard the fruit fly, *Drosophila melanogaster* (*Drosophila*), has served as an important system to study the consequence on nervous system function of mutations in genes associated with cognitive [26] and psychiatric [27] disorders, including several ASD-associated genes, such as *Neurexin* [28], *Shank* [29], *Drosophila fragile X mental retardation gene* [30], and *neuroligin 2* [31, 32].

The *transient receptor potential 6 channel* (*TRPC6*) gene is considered a candidate for ASD (SFARI 2, strong candidate class) based on the identification of a non-syndromic ASD individual that carried a de novo balanced translocation that disrupts one copy of the *TRPC6* gene, located on chromosome 11q22, together with the enrichment of predicted pathogenic *TRPC6* variants in ASD cases as compared to controls [33]. Disruptive TRPC6 variants may act together with other genetic factors that contribute to the ASD phenotype, as incomplete penetrance was observed in some families [33]. *TRPC6* encodes a voltage-independent, Ca^2+^-permeable, cation channel, and although knowledge of its function in the human brain is only rudimentary [34], the consequences on neuronal function of disrupting the *TRPC6* gene were recently investigated in induced pluripotent stem-cell (iPSC)-derived neuronal cells and dental pulp cells obtained from the initial patient [33]. In these cells a reduction in *TRPC6* expression was consistently observed, which caused a decrease in the phosphorylation of CREB (a transcription factor activated upon Ca^2+^ influx through *TRPC6*), and, in turn, resulted in the deregulation (increase or decrease) of target gene expression. In addition, reduced Ca^2+^ influx was observed in the patient’s neuroprogenitor cells, and the resulting neurons exhibited decreased excitatory synapses, and reduced arborization and dendritic spine density [33]. *TRPC6* knockdown (through the use of short hairpin RNA, or shRNA) caused similar changes in isogenic control cells. Interestingly, all the cellular phenotypes expressed by the patient’s neuronal cells were rescued by hyperforin treatment (a TRPC6 agonist). Together, these results support the haploinsuficiency model, and suggest that that the defects observed in cells derived from the patient, and thus, possibly also those expressed by the patient, are due to a deficiency of TRPC6 [33].

Here, we extend our analysis of the role of *TRPC6* in ASD. First, in a 10-year follow-up examination, we provide a more detailed clinical description of the index translocation carrier. We then describe new loss-of-function mutations in *TRPC6* carried by ASD patients from unrelated families. Then, using the fruit fly, *Drosophila*, we examine the functional consequence of disrupting the *trp-gamma* gene (*trpγ*; CG5996), the fly gene most similar to *TRPC6*, on the control of behavior. Using this model organism, we found that null *trpγ* mutant flies exhibit several behavioral anomalies that mirror those seen in ASD patients, including defective social interactions, hyperactivity (in both young and older flies), impaired learning and memory, and deficits in sleep homeostasis. Some defects, notably in sleep, were more severe in males than in females and became attenuated with age. Interestingly, we found that the chemical hyperforin (the primary active phytochemical of St. John’s wort) alleviated many of the defects of *trpγ* mutant flies, consistent with the effects reported for *TRPC6*-deficient neuronal cells [33]. Overall, our data suggest that *TRPC6* is an ASD susceptibility gene and that its role in the control of behavior can meaningfully be investigated in *Drosophila*.

## Materials/subject and methods

### Identification of new *TRPC6* mutant alleles in ASD individuals

We searched multiple microarray and whole genome databases for CNVs and loss-of-function variants in *TRPC6* by mining internal microarray CNV databases of published and unpublished samples of various neurodevelopmental disorders (*n* = 5328) including ASD, ADHD, OCD, schizophrenia, impulsivity, and cerebral palsy, which is hosted at the Centre for Applied Genomics in the Hospital for Sick Children in Toronto [11]. We also analyzed the whole genome sequence of autism cases in cohorts of MSSNG (*n* = 5102) and of the Simon Simplex Collection (*n* = 2419). The methods for identifying CNVs and sequences level variations from whole genome are described in details in Zarrei et al. [11], Yuen et al. [17], and Trost et al. [22]. We also searched publications reporting on mutations in *TRPC6*. We did not include here missense variants as their effects on gene function are difficult to predict; such variants have been associated with FSGS2 (Glomerulosclerosis, focal segmental 2; #MIM603965) caused by a gain-of-function mechanism and in a few cases by a dominant negative effect [35, 36].

### Fly rearing and stocks

*Drosophila* strains were raised on standard cornmeal media and, unless noted, were maintained at room temperature (20–22 °C) on a 12 h light:12 h dark (12 L:12D) light:dark (LD) cycle. Young flies were defined as those <10 days old and old flies as >30 days old [37]. We used wild type *Canton-S* and *white*^*1118*^ (*w*^*1118*^) flies as control genotypes, as appropriate. *trpγ*^*1*^ and *trpγ*^*G4*^ (an insertion of the GAL4 transcription factor into the *trpγ* gene, CG5996) are null *trpγ* alleles and were kindly provided by Craig Montell (University of California Santa Barbara, California, USA), as were UAS-*trpγ*, and a *trpγ[+]* transgenic rescue line [38]. UAS-RNAi lines for *trpγ* were obtained from the Vienna *Drosophila* RNAi Center, Vienna, Austria (VDRC) (stock VDRC105280) and the Bloomington *Drosophila* Stock Center at Indiana University, Bloomington, USA (BL) (stock BL31299). Preliminary tests showed that the results obtained using the VDRC UAS-*trpγ* RNAi line were more severe and similar to those obtained with the *trpγ* null alleles. For this reason, all results reported here used RNAi line 105280 from VDRC. The *elav*-GAL4 driver (stock BL458), flies bearing a chromosomal deletion that uncovers *trpγ* (*Df(2* *L)ED1109*; stock BL8945) and reporter UAS-mCD8::GFP (stock BL5137), were obtained from the Bloomington *Drosophila* Stock Center. For hyperforin treatments we placed adult flies on apple juice-agar media containing 10 µM hyperforin (H1792, Sigma-Aldrich) for 96 h prior to testing.

### Behavioral assays

We used a number of well-established quantitative behavioral assays to test *Drosophila* neural function, including courtship, anxiety-like behavior, learning and memory, circadian rhythmicity, locomotor activity, and sleep and sleep homeostasis, some of which have previously been used to investigate the behavioral capabilities of flies bearing mutations in homologs of genes associated with ASD [39].

### Courtship

Courtship behavior was quantified as described in McBride et al. (1999) [40]. Briefly, males were collected upon emergence and kept individually in food vials until tested. Courting pairs (individual male + a virgin female) were placed in a standard “courtship chamber” and their behavior video recorded for 10 min. The courtship index (CI; proportion of time spent by the male in active courtship during these 10 min or until copulation, whichever happens first) and the timing of each stereotyped element of courtship behavior (following, tapping, wing vibration, licking, and copulation attempts) was measured [41, 42].

### Anxiety-like behavior

Anxiety-like behavior was assessed using an open field assay as described in Besson and Martin (2005) [43]. Briefly, flies were placed individually in a custom-made arena (4 × 4 cm and 3.5 mm high), and their behavior video recorded during 10 min. The ANY-maze tracking software (http://www.anymaze.co.uk/) was then used to measure average locomotion speed, total distance traveled, number of entries to the central zone of the arena, and time spent in the central vs. the peripheral zone of the arena.

### Learning and memory

The courtship-conditioning assay was used to measure learning and short-term memory [44, 45]. For these tests males were collected on the day of emergence and aged individually in food vials at 25 °C under 12/12 h LD cycle for 4–7 days (for young flies) and 40–72 days (for old flies). Courtship conditioning was carried out essentially as described in Ejima and Griffith et al. (2011) [44]. This test is based on the fact that male flies eventually stop courting a mated (unreceptive) female. This learned behavior also reduces his subsequent courtship toward a virgin female; the perdurance of this reduced courtship can then be used to measure short-term as well as long-term memory [44]. To ease the reading of the figures so that better learning and memory are shown as higher scores, the results shown here were calculated as 1 minus the values recorded [46].

### Circadian rhythmicity

Standard procedures were used to determine the status of the circadian clock. Briefly, 1–3 day old adult flies were entrained to a 12 L:12D LD regime for 3 days, placed individually in Trikinetics monitors (Trikinetics, USA), and their activity measured every 30 min for 7–10 days under conditions of constant darkness. Resulting records were analyzed using Matlab (MathWorks, Inc., Natick, USA) and the appropriate Matlab toolbox [47].

### Locomotor activity

The activity of 1–3 day old adult flies was recorded for 7–10 days under a 12 L:12D LD regime using the Trikinetics locomotor activity monitors described above and used to calculate average day, night, morning, and evening activity. The “morning” and “evening” intervals were defined as the 3 hours after lights on and the 3 hours before lights off, respectively.

### Sleep

A sleep episode in *Drosophila* is defined as a period of immobility of >5 min duration [48], and was assayed essentially as described in Shaw et al. (2000) [49]. For this, the same Trikinetics monitors used for locomotor activity were used except that activity was measured every minute. The total duration of sleep, the average number of sleep episodes, and their duration (sleep consolidation) under 12L:12D LD conditions were derived from these records using a Matlab-based analysis package [50].

### Sleep homeostasis/rebound

Flies were sleep-deprived during a single 12 h dark period of a 12L:12D LD regime using a standard sleep disruption protocol involving mechanical agitation (2 s every 10 s) [51]. Sleep homeostasis (“sleep rebound”) was then determined by comparing the sleep parameters during the 24 h following sleep deprivation to those of the average for the 2 days prior to the sleep deprivation episode.

### *Drosophila* lifespan assay

We performed this assay essentially as described in Linford et al. (2013) [52]. After emergence, male and female flies of each genotype were kept separately at 25 °C in groups of 20 in plastic vials with standard food, and transferred every 2 days to fresh food vials. The number of dead flies was then counted every 2 days. A minimum of three replicates was performed per genotype.

### Immunostaining

The pattern of *trpγ* expression was obtained by crossing the *trpγ*^*G4*^ GAL4 driver to the UAS-mCD8::GFP reporter. Tissues were fixed in 4% buffered paraformaldehyde for 1 h at room temperature and rinsed in PBS + 0.3% Triton-X (Sigma-Aldrich, USA). They were then rinsed in PBS and imaged directly (adult CNS and legs) or first processed for anti-GFP immunoreactivity (larval and pupal CNS) using a rabbit anti-GFP antiserum (1:1,000; A6455, Invitrogen, CA, USA) and anti-rabbit IgG Alexa Fluor 488 secondary (Invitrogen, MA, USA).

### Statistical analyses

Statistical comparisons between genotypes were carried out using Prism 9.0 (Graph Pad Software Inc, CA). *t*-tests, and one-way ANOVA followed by Tukey’s post hoc multiple comparison analyses, were used for normally distributed data (Supplementary Table 2). For analyses of behavioral rhythmicity, values were compared by one-way ANOVA, followed by Tukey’s post hoc multiple comparison analyses. Fisher’s exact test was used to analyze contingency tables. Differences in survival were analyzed using Kaplan–Meier survival plots; log-rank analysis was carried out using the OASIS online survival analysis package [53]. Statistical significance is indicated in each figure using: “****”:*p* < 0.0001; “***”:*p* < 0.001; “**”:*p* < 0.01; “*”:*p* < 0.05. In addition, Supplementary Table 2 contains the exact values for all comparisons. The number of animals (N) used for each experiment and genotype is indicated on each figure. The minimum number used was 10, but in most cases exceeded 20. No sample size estimate was calculated to detect a pre-specified effect and no results were excluded.

## Results

### Case re-evaluation

The index ASD patient was first referred to our center (Centro de Estudos do Genoma Humano e Células Tronco, University of Sao Paulo, Brazil) for genetic investigation as a non-verbal ASD 5 year old patient, who also suffered from severe intestinal constipation and had sleeping problems. He was found to carry a de novo balanced translocation between the chromosome 3 and 11, 46, XY t(3;11)(p21;q22), disrupting the *TRPC6* and *VPRBP* genes [33]. No abnormalities were found on audiometric testing, electroencephalography (EEG), or cerebral magnetic resonance imaging (MRI) scan, performed when he was 4–5 years old [33]. He was then re-assessed in 2020, at 17 years of age (Supplementary Table 1) and had not shown any improvement in ASD behavioral symptoms, language, or learning. However, he had suffered an epilepsy episode at age 13, and has been taking carbamazepine. His most recent EEG showed epileptiform activity and his MRI showed a minor hippocampal asymmetry (left side is <10% smaller than right). He also suffered from anxiety behavior and hyperactivity, which were being treated with Sertraline and Risperidone, respectively.

### Identification of new ASD cases bearing mutations in *TRPC6*

In order to further investigate the link between disruptions in *TRPC6* and ASD, we searched available databases for additional ASD patients bearing rare loss-of-function *TRPC6* alleles. We found no CNVs in *TRPC6* amongst published and unpublished cases. However, we found eight cases (of which five were previously unreported) carrying predicted loss-of-function mutations (frameshift deletion or insertions, stop-gain, and canonical splice-site mutations) impacting *TRPC6* (Table 1). Except for a splice-site variant, all others are predicted to create premature stop codons in the N-terminal domain of the TRPC6, located upstream of the transmembrane domain. The in silico prediction of the consequences on TRPC6 expression caused by the change in splice site (patient 2-1280-003, Table 1) are unclear, but could result in the skipping of exon 4, which would lead to the loss of 55 amino acids, including the beginning of the transmembrane domain. Three of six cases with available genomic information also carried additional clinically relevant CNVs or canonical splice-site mutations; however, none of them have been associated with monogenic forms of ASD (Table 1).

**Table 1:** Phenotypic description of individuals with *TRPC6* variants^a,b^.

GENERAL INFORMATION	Participant ID	F2749-1	2-1280-003	3-0817-000	7-0089-003	MSSNG00188-003	MSSNG00070-003	SSC04382	iHART1937	iHART1939
	Date of Birth (DOB - MM/YY);	07-2002	09-2008	10-2011	06-2007	NA	NA	NA	NA	NA
	Sex	Male	Female	Female	Female	Male	Male	Male	Male	Male
Genetic data	Variant genomic position [hg38]	t[3;11] [p21;q22]	g.101488935 A > G	g.101489024_101489026delinsGG	g.101504768_101504792dup	g.101491611dup	g.101583364delG	g.101583497 G > A	g.101489023delT	g.101489023delT
	cDNA variant	NA	c.1293 + 2 T > C	c.1204_1206delinsCC	c.177_201dup	c.1073dup	c.140delC	c.7 C > T	c.1207delA	c.1207delA
	Protein change	NA	p.?	p.T402Pfs	p.R68fs	p.H358fs	p.P47Rfs*33	p.Q3*	p.M403Wfs*33	p.M403Wfs*33
	Inheritance	de novo	Maternal	Maternal	Paternal	Maternal	Unknown; not maternal	Paternal	Maternal	Maternal
	gnomAD frequency	Not applied	0	0	0	0.00000409	0	0.0000956	0.00000398	0.00000398
Development	ASD evaluation	DSM-IV and CARS (severe autism)	ADI/ADOS	ADOS	ASD	ADI/ADOS	ASD	ASD	ADI-R	ASD by ADI-R
	Global Ability	Low	Average	NA	NA	NA	NA	NA	NA	NA
	Intellectual disability; IQ	Yes (unable to learn how to read or to recognize numbers)	Wechsler; Average IQ (105 standard score)	NA	Yes	NA	NA	NA	NA	NA
	Adaptive Behavior	NA	VABS; very low daily adaptive skills (74 standard score)	VABS; impaired; extremely low daily adaptive skills (69 standard score)	NA	NA	NA	NA	NA	NA
	Socialization	Low	Extremely low (68 standard score) based on VABS	Extremely low (65 standard score) based on VABS	NA	NA	NA	NA	NA	NA
	Age of walking	18 months	12 months	NA	NA	NA	NA	NA	NA	NA
	Age of first words	Non-verbal	45 months	NA	NA	NA	NA	NA	NA	NA
Development (continued)	Language ability	None	Average receptive vocabulary skills; low average overall language skills	Language skills limited; fall in very low range for communication; based on ADOS and VABS	NA	NA	NA	NA	NA	NA
	Behavioral issues	Repetitive behaviors, resistant to change routine	Repetitive behaviors, resistant to change	NA	NA	NA	NA	NA	NA	NA
	Seizures/epilepsy	Yes	No	No	No	NA	NA	NA	NA	NA
	Feeding issues	No	NA	NA		NA	NA	NA	NA	NA
	Other neuropsychiatric or neurodevelopmental disorder	Hyperactivity and anxiety behavior	NA	NA	Auditory Processing Disorder	NA	NA	NA	NA	NA
Other	Eye abnormalities/vision problems	No	NA	NA	NA	NA	NA	NA	NA	NA
	Ears/hearing loss	None	NA	NA	Sensorineural hearing loss	NA	NA	NA	NA	NA
	Other Dysmorphologies/congenital abnormalities	None	NA	NA	Dysmorphic, unspecified	NA	NA	NA	NA	NA
	Other genetic variants	Not identified in WES^37^	Variant is also seen in unaffected brother	Additional mutation: 7p22.3, 506 kb DUP, including *BRAT1*, unknown origin	Not identified	Additional mutation: Xq13.3-q21.1, 9.4 Mb DUP including *ABCB7, FGF16, ATRX, ATP7A, PGK1, TBX22, BRWD3, POU3F4*; maternal	Not identified	Not identified	Variant is also seen in affected brother	Variant is also seen in affected brother
	Family history	No family history among 1st related relatives	NA	Family history of ASD, ADHD, depression and learning disability	Family history of diabetes, and schizophrenia	NA	NA	NA	NA	NA
	Source	Griesi-Oliveira et al. ^37^	MSSNG^21^	MSSNG^21^	MSSNG^21^	MSSNG^21^	MSSNG^21^	Griesi-Oliveira et al. ^37^	Ruzzo et al.^104^	Ruzzo et al.^104^

^a^Variants were mapped to RefSeq transcript NM_004621.5 isoform of *TRPC6* and are based on Human Genome Build GRCh38. For patient F2749-1, the translocation breakpoint was mapped in VPRBP (NM_001387579.1) and TRPC6 (NM_004621). All cases in this table were from MSSNG except for SSC04382, which is from Simon Simplex Collection (SSC) and has also been reported in Griesi-Oliveira et al. (2014) [33]. All variants are heterozygous. All variants from MSSNG were confirmed using Sanger sequencing. We did not have DNA from the SSC case (SSC04382) to perform a Sanger validation.

^b^Except for the variant c.140delC predicted as likely pathogenic (ACMG: PVS1, PM2), all the others were predicted to be pathogenic (ACMG: PVS1, PM2, PP3).

### *trpγ*, the fly gene most similar to human *TRPC6*

At the cellular level, iPSC-derived neuronal cells from the initial ASD patient exhibit reduced Ca^2+^ influx, as well as decreased excitatory synapses, and reduced arborization and dendritic spine density [33]. However, little is known about how mutations in *TRPC6* affect behavior. In order to further our understanding of the impact of mutations in this gene on nervous system function we turned to the model system, *Drosophila*, which has been extensively used to research the genetic bases of behaviors [32, 41].

We used DIOPT [54] followed by BLAST [55] analyses in order to identify *trpγ* (CG5996) as a potential *Drosophila* homolog of *TRPC6*. This gene was selected because it is the fly gene with the highest overall score and homology to *TRPC6* (38% identity, 57% similarity, covering 86% of the sequence) and functional domains (Supplementary Fig. 1). In addition to previously reported expression of *trpγ* in the proprioceptive organs of the fly [38], we found that *trpγ* is expressed in the fly CNS (Supplementary Fig. 2g), suggesting that the behavioral defects described below may be due to functions subserved by this gene in central neurons (Supplementary Fig. 2a–f). We also found that *trpγ* expression is extremely low and sparse up until the start of metamorphosis, suggesting that defects in *trpγ* signaling may primarily affect the function, and not the development, of the relevant neuronal circuits.

We used a number of well-established quantitative behavioral assays in order to understand the consequences of mutations in *trpγ* on neural function. For all behavioral assays used, animals mutant for *trpγ* were mostly *trans*-heterozygous for two different null *trpγ* alleles. The first, called here *trpγ-*GAL4, carries an insertion of the GAL4 transcription factor just downstream of the *trpγ* translation start site and includes a 547 base pair deletion extending 3′ from the start codon [38]; this strain also allows gene expression to be driven in a pattern that reflects the temporal and spatial expression of the *trpγ* gene (cf. Supplementary Fig. 2, below). The second null *trpγ* allele, called *trpγ*^*1*^, carries a 180 base pair deletion that removes the carboxyl-terminal portion of the sixth transmembrane domain as well as the highly conserved TRP box.

### *trpγ* function is required for normal courtship behavior

We first examined *Drosophila* courtship behavior as it involves a sequence of stereotyped routines that require that the male fly pay attention to the female and adjust his behavior depending on her responses to his advances [41, 42]. During courtship the male first orients toward the female, then follows her while producing a species-specific “courtship song” by vibrating one of his wings [41, 42]. Depending on the female’s receptivity and the male’s engagement, the additional steps of the fixed courtship sequence are then executed by the pair, culminating in copulation. Should the female be unreceptive or the male not be fully engaged, any step of the sequence can be extended or the sequence aborted entirely if the female rejects the male. Thus, courtship provides a sensitive assay for detecting abnormalities in a behavior that requires inter-individual communication [39].

**Fig. 1:**
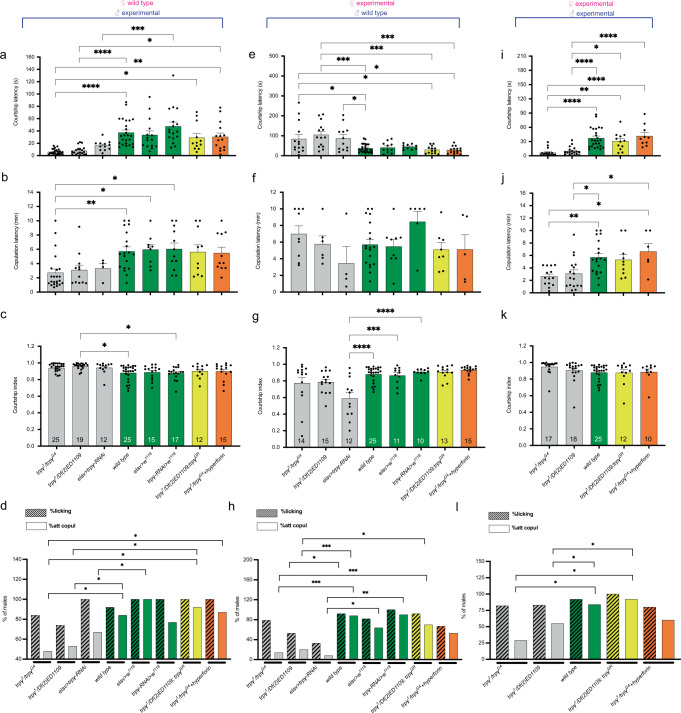
*trpγ* mutant males express increased courtship drive. Courtship latency (time to initiate courtship; **a**, **e**, **i**), copulation latency (time to copulate; **b**, **f**, **j**), courtship index (**c**, **g**, **k**) and courtship ethogram (**d**, **h**, **l**) for pairs in which males of different genotypes were tested with wild type (*Canton-S*) females (**a**–**d**), pairs in which females of different genotypes were tested with wild type (*Canton-S*) males (**e**–**h**), and pairs in which both males and females of different (but matched) genotypes were tested (**i**–**l**). Genotypes for (**a**–**k**) are indicated along *X*-axis of (**c**, **g**, **k**), respectively; results are color-coded such that gray columns correspond to transheterozygous null *trpγ* mutant alleles (or CNS specific *trpγ* knockdown), green columns correspond to relevant control genotypes, yellow columns correspond to genomic rescue of the transheterozygous null *trpγ* mutant alleles, and orange columns correspond to transheterozygous null *trpγ* mutant alleles fed hyperforin. For figures (**d**, **h**, **I**) the percentage of males that execute licking and attempted copulation behaviors are indicated in striped and non-striped columns, respectively. Results are shown as means ± s.e.m.; numbers within bars of (**c**, **g**, **k**), corresponds to the number of flies tested. Data were analyzed by a one-way ANOVA, followed by Tukey´s post hoc multiple comparison analyses where “****”: *p* < 0.0001; “***”: *p* < 0.001; “**”: *p* < 0.01; “*”: *p* < 0.05. Only the most important statistical differences are shown in the figure. See Supplementary Table 2 for exact values for all comparisons.

In order to evaluate the role of *trpγ* in the control of courtship behavior, we first examined the behavior of males trans-heterozygous for the *trpγ* null alleles (*trpγ*^*1*^ and *trpγ*^*G4*^) or for *trpγ*^*1*^ and a chromosomal deletion that uncovers *trpγ* (*Df(2)ED1109*), as well as of males in which *trpγ* was specifically knocked down in all neurons. As shown in Fig. 1d and Supplementary Fig. 3, male flies performed the entire courtship sequence in the correct order, culminating in copulation (see exact results of statistical analyses for this and all figures in Supplementary Table 2). However, when paired with a wild type (*Canton-S*) female, *trpγ* mutant males initiated courtship with significantly shorter latency (Fig. 1a; gray bars, in this and all figures) and achieved copulation significantly faster (Fig. 1b) and with fewer copulation attempts (Fig. 1d) than did males of the relevant control genotypes (green bars, in this and all figures). These reduced latencies did not affect the CI, which is a measure of the attractiveness of a female during the time of assay (Fig. 1c). Importantly, normal courtship behavior was restored when a wild type copy of *trpγ* (*trpγ* [+]) was introduced in a hemizygous mutant background (yellow bars, in this and all figures), demonstrating that these behavioral defects map to the *trpγ* gene. Interestingly, normal behavior was also rescued by feeding the mutant males hyperforin (10 μM) for 4 days prior to testing (orange bars, in this and all figures), reminiscent of the rescue of morphology and function observed for *TRPC6*-deficient neuronal cells [33].

We then evaluated the courtship of wild type (*Canton-S*) males toward virgin *trpγ* mutant and knockdown females (Fig. 1e–h) and found that males took much longer to initiate courtship when paired with these females (Fig. 1e), and although they then showed normal latency to copulation (Fig. 1f), they expressed a lower overall CI (Fig. 1g). This reduced courtship drive is also evident in the reduction in the percentage of males that executed the different steps of the behavioral sequence, including following/tapping, wing vibration, licking, and attempted copulation (Fig. 1h and Supplementary Fig. 3b). Again, normal behavior was restored when a wild type copy of *trpγ* was introduced in a hemizygous mutant female, and also when she was fed hyperforin for 4 days prior to testing (Fig. 1e–h and Supplementary Fig. 3b). Thus, these results show that normal males express lower courtship drive toward *trpγ* mutant females revealing an important role for females in the control of the courtship behavior [56]. Finally, we found that when both partners were mutant or expressed a knockdown of *trpγ* function (Fig. 1i–l) mutant males behaved as they did when paired with wild type females and expressed a shorter latency to initiate courtship (Fig. 1i), copulated sooner (Fig. 1j), with lower percentage of copulation attempts (Fig. 1l and Supplementary Fig. 3c), yet expressed a normal CI (Fig. 1k). These defects were also rescued by introducing a wild type copy of *trpγ* and by feeding hyperforin to the male. Overall, our analysis of courtship behavior suggests that the *trpγ* gene is involved in regulating courtship drive in males, with mutant males expressing an increased urgency to copulate, whereas it seems to regulate courtship attractiveness in females.

### *trpγ* mutant flies are hyperactive

**Fig. 2:**
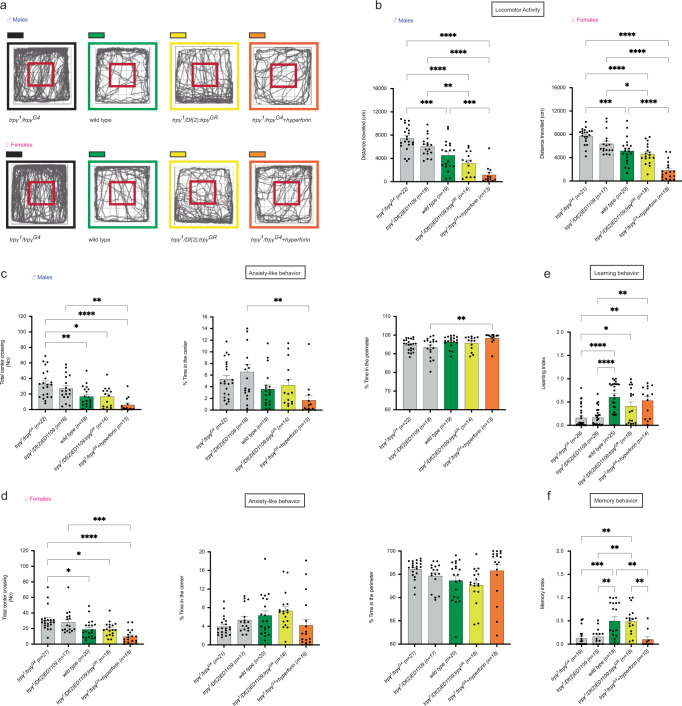
Mutations in *trpγ* cause hyperactivity and affect learning, and memory. **a**–**d** Behavior of adult flies in open field test. **a** Representative traces of individual flies; **b**–**d** Quantification of behavior; **b** Total distance traveled; **c**, **d** Total number of crossings into central area of arena (left), and Proportion of time spent in center (center) and perimeter of arena (right) for males (**c**) and females (**d**); Learning (**e**) and Memory (**f**) of males in courtship conditioning assay. Genotypes are indicated along the *X*-axes and color-coded as described in Fig. 1, with the number of animals tested indicated in parenthesis. Statistical significance for (**b**–**f**) is coded as described in Fig. 1. Only the most important statistical differences are shown in the figure. See Supplementary Table 2 for exact values for all comparisons.

ASD individuals have high prevalence of neurological comorbidities, including attention deficit or hyperactivity and anxiety symptoms [57]. Similarly to rodents [58], in *Drosophila*, the preference of adult flies for walking in the center vs. along the wall of an open field has been used to evaluate anxious behaviors [43, 59]. Using this assay, we found that *trpγ* mutant male and female flies were hyperactive, which is evident in the traces of individual flies (Fig. 2a; traces boxed in gray) and is reflected in the increased total distance travelled compared to that of controls (Canton-S) (Fig. 2b; gray vs. green bars); they also expressed significantly greater number of entries to the central zone of the arena. Normal levels of locomotor activity were restored by incorporating a wild type copy of *trpγ* (Fig. 2c, d, yellow bars) and also by feeding hyperforin for 4 days prior to testing (Fig. 2c, d; orange bars). Nevertheless, neither males nor females spent comparatively more time in the peripheral vs. central part of the arena than did controls (Fig. 2c, d), suggesting that mutations in *trpγ* do not affect anxiety levels.

### Loss of *trpγ* function impairs learning and memory

ASD individuals typically show cognitive disabilities, memory reduction, and self-focused attention [60], whose severity is correlated with the severity of their autism disorder [61]. Here we used the “courtship conditioning” assay to evaluate *Drosophila* learning and memory [44, 45]. This assay is based on the fact that a mated female rejects the advances from a male fly and, as a result, the male eventually stops courting her. This learned behavior also reduces his subsequent courtship toward any female (including a virgin female), and the perdurance of this reduced courtship can then be used to measure short-term as well as long-term memory. We found that *trpγ* mutant males displayed significantly lower levels of learning (Fig. 2e) and memory (Fig. 2f) compared to controls (*Canton-S*). Both defects were rescued by introducing a wild type copy of *trpγ*. Feeding hyperforin for 4 days prior to performing these assays rescued the learning defects (Fig. 2e), but did not improve memory levels (Fig. 2f).

### Loss of *trpγ* function impairs sleep and sleep homeostasis

**Fig. 3:**
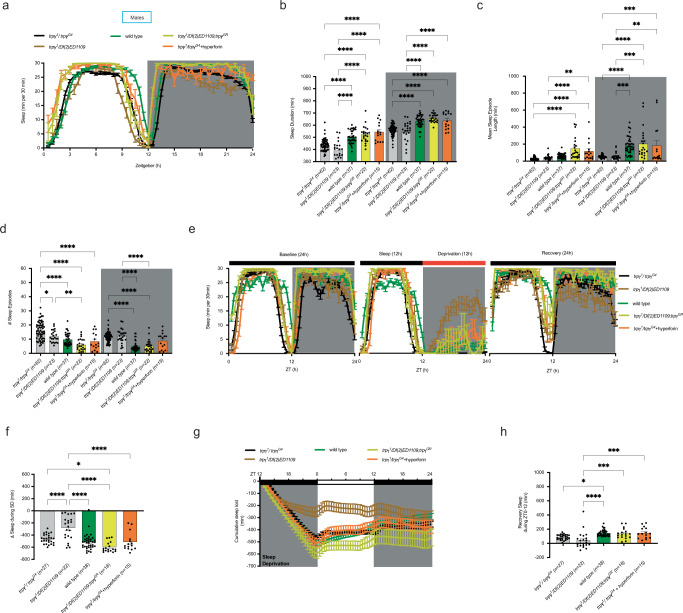
Mutations in *trpγ* affect sleep and sleep homeostasis of male flies. **a**–**d** Sleep behavior under L:D regime. Average: Sleep profiles (**a**); Sleep duration (**b**); Length of sleep episodes (**c**), and Number of sleep episodes (**d**). **e**–**h** Sleep recovery following 12 h sleep deprivation (SD). **e** Average sleep profiles on day prior to SD, on day of SD and on day following SD. **f** Average sleep loss during SD; (**g**, **h**) Average: trace of sleep following SD (**g**) and of sleep recovery during first 12 h following SD (**h**). Genotypes are indicated along the *X*-axes and color-coded as described in Fig. 1, with the number of animals tested indicated in parenthesis. We used *white* mutant flies (*w*^*1118*^) as control genotype for all sleep assays. Statistical significance for (**b**–**d**, **f**, **h**) is coded as described in Fig. 1. Only the most important statistical differences are shown in the figure. See Supplementary Table 2 for exact values for all comparisons.

Sleep problems are often observed in ASD individuals including the carrier of the *TRPC6* ASD index case (Supplementary Table 1), which can worsen the ASD core symptoms and the disorder prognosis [62–64]. *Drosophila* exhibit periods of sleep-like states, which share many features of mammalian sleep [65]. To examine the role of *trpγ* in *Drosophila* sleep, we assessed sleep behavior in *trpγ* mutant males and females under a 12 h light:12 h dark LD regime. We found that *trpγ* mutant males expressed a significant decrease in total sleep duration, and shorter and more frequent sleep episodes (Fig. 3a–d; gray traces and bars) than did controls (*white*^*1118*^, *w*^*1118*^; green traces and bars). Normal sleep was restored by introducing a wild type copy of *trpγ* in a hemizygous mutant background (Fig. 3a–d; yellow traces and bars). It was similarly restored by feeding the flies hyperforin for 4 days prior to initiating the sleep assay (Fig. 3a–d; orange traces and bars). Interestingly, we found that the decrease in sleep duration persisted under conditions of constant darkness (Supplementary Fig. 4a–b).

As in mammals, the timing and the amount of *Drosophila* sleep are governed by a circadian mechanism as well as by a homeostatic mechanism that counts the number of hours awake [65]. To investigate the status of the sleep homeostat in *trpγ* mutant *Drosophila*, we sleep-deprived flies by mechanical stimulation for 12 h during the night (Fig. 3e, Supplementary Fig. 5a–c) then measured the amount of sleep that was recovered during the following day. As expected, males of all genotypes lost sleep during sleep deprivation (Fig. 3f), yet *trpγ* mutant males then recovered significantly less lost sleep than did control flies (Fig. 3g–h). This reduced sleep homeostasis was restored by introducing a wild type *trpγ* transgene as well as by feeding hyperforin prior to testing. Unlike the defects observed for sleep and sleep homeostasis, *trpγ* mutant males expressed normal circadian rhythms of locomotor activity under conditions of constant darkness (DD) (Supplementary Fig. 6a–f), indicating that in males, mutations in *trpγ* do not affect the circadian clock.

**Fig. 4:**
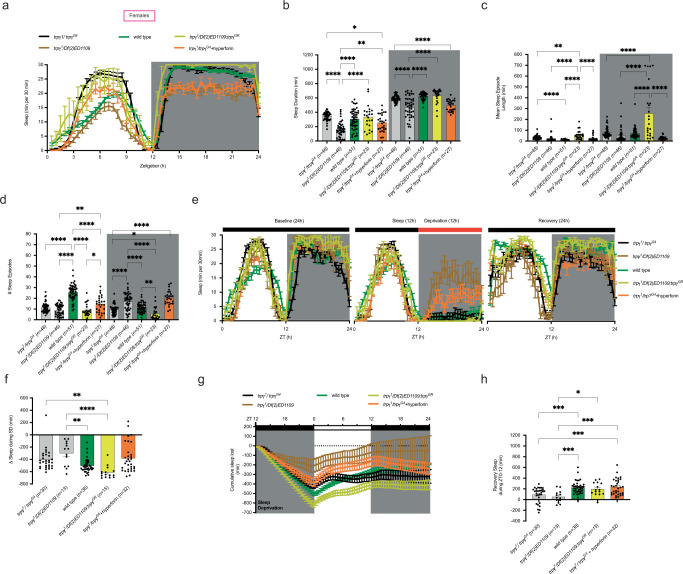
Mutations in *trpγ* cause inconsistent defects in sleep but affect sleep homeostasis of female flies. **a**–**d** Sleep behavior under L:D regime. Average: Sleep profiles (**a**); Sleep duration (**b**); Length of sleep episodes (**c**) and Number of sleep episodes (**d**). **e**–**h** Sleep recovery following 12 h sleep deprivation (SD). **e** Average sleep profiles on day prior to SD, on day of SD and on day following SD. **f** Average sleep loss during SD; (**g**, **h**) Average: trace of sleep following SD (**g**) and of sleep recovery during first 12 h following SD (**h**). Genotypes are indicated along the *X*-axes and color-coded as described in Fig. 1, with the number of animals tested indicated in parenthesis. Statistical significance for (**b**–**d**, **f**, **h**) is coded as described in Fig. 1. Only the most important statistical differences are shown in the figure. See Supplementary Table 2 for exact values for all comparisons.

ASD symptoms and the impact of risk factors can differ in men and women [66], and, interestingly, we found that, unlike males, *trpγ* mutant female flies did not show consistent differences in total sleep compared to controls (Fig. 4a, b; gray vs. green traces and bars). Nevertheless, sleep recovery was similarly reduced compared to that of controls (Fig. 4g, h), and was also rescued by introducing a wild type *trpγ* transgene (Fig. 4g, h, yellow traces and bars) as well as by feeding hyperforin before testing (Fig. 4g, h, orange traces and bars). We also evaluated a possible role for *trpγ* in the circadian control of sleep and under conditions of constant darkness found that *trpγ* mutant females expressed a decrease in sleep duration during the subjective day (Supplementary Fig. 7a–b). As in males, circadian rhythmicity of locomotor activity was normal (Supplementary Fig. 8a–f), indicating that the mild sleep defects expressed by females were due to alterations in sleep and sleep regulation, not to defects in the circadian clock itself.

### Effects of age on *trpγ* mutant flies

**Fig. 5:**
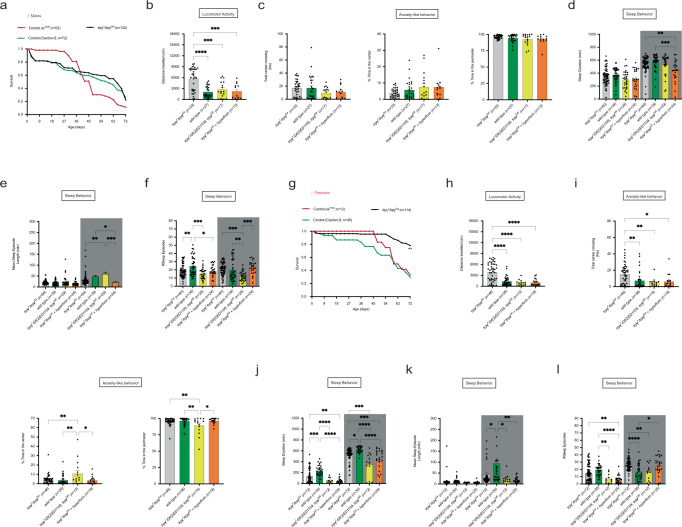
Impact of mutations in *trpγ* on longevity, hyperactivity, and sleep. **a**–**f** Behavior of aged males. **a** Average longevity of *trpγ* mutants and controls. **b**, **c** Anxiety-like behavior. Average: Distance traveled (**b**); Time (left) and proportion (right) of time in periphery of arena (**c**). **d**–**f** Average: Sleep duration (**d**); Duration of sleep episodes (**e**), and Number of sleep episodes (**f**). **g**–**l** Behavior of aged females. **g** Average longevity of *trpγ* mutants and controls. **h**, **i** Anxiety-like behavior. Average: Distance traveled (**h**); Time (left) and proportion of time (right) in periphery of arena (**i**). **j**–**l** Average: Sleep duration (**j**); Duration of sleep episodes (**k**), and Number of sleep episodes (**l**). Genotypes are indicated along the *X*-axes and color-coded as described in Fig. 1, with the number of animals tested indicated in parenthesis. Statistical significance for (**b**–**d**, **f**, **h**) is coded as described in Fig. 1. Only the most important statistical differences are shown in the figure. See Supplementary Table 2 for exact values for all comparisons.

The study of ASD has mostly focused on children and adolescents, and while some studies are collecting longitudinal data, little is known about the impact of aging on the severity of this disorder, and how different genes contribute in this process [67, 68]. We did not observe differences in the survival [52] of *trpγ* mutant vs. control males (Fig. 5a). Although both groups of aged flies expressed a sharp decline in their levels of locomotor activity (Compare Fig. 5b with Fig. 2b), aged mutant male flies remained hyperactive when compared to controls (Figs. 2b and 5b; gray vs. green bars). And, as occurred in younger flies, this defect was rescued by introducing a wild type copy of *trpγ* (Fig. 5b; yellow bars) and by feeding hyperforin for 4 days prior to assay (Fig. 5b; orange bars). Interestingly, and unlike younger flies, older *trpγ* mutant males did not express consistent sleep defects (Compare Fig. 3b–d with Fig. 5d–f). In parallel experiments, we observed that, in contrast to males, *trpγ* mutant female flies showed a higher survival than controls. Yet, similarly to males, older mutant females were much more active than their normal counterpart (Figs. 2d and 5h). Neither older male (Fig. 5c) nor older female (Fig. 5i) mutant animals expressed the hallmarks of anxiety-like behaviors.

In summary, we found that *trpγ* mutant flies exhibited a number of behavioral anomalies that mirror those seen in ASD patients (Table 1; Supplementary Table 1), including defective social interactions, hyperactivity, impaired learning and memory, and deficits in sleep homeostasis. Interestingly, some of these behavioral defects differed between genders (e.g., in sleep) and were differentially affected by age (e.g., sleep in males vs. hyperactivity).

## Discussion

Here we performed a 10 years follow-up clinical analysis of an ASD individual who carries a disruption in the *TRPC6* gene [33], and report that he is still non-verbal and presented one episode of epilepsy. We also identified eight additional patients diagnosed with ASD that inherited putative loss-of-function mutations in *TRPC6* that are classified as possibly pathogenic or as pathogenic according to the ACMG (Table 1). Notably, all the TRPC6 variants so far associated with ASD are located in the N-terminal domain of the protein, which could disrupt the transmembrane domain of TRPC6, in agreement with the haploinsufficiency model [33]. An inspection of the distribution of the TRPC6 variants in the Gnomad revealed a lower frequency of LoF variants in the N- terminal (24/74,156 genomes; variants downstream amino acid 500) as compared to those found in the MSSNG/SSC databases (7/7521 genomes; Table 1; *p* = 0.018; odds ratio: 2.95 [1.07–7.07]). In addition, none of these patients carried pathogenic lesions that might have explained their phenotype. Language and social skills were compromised in all cases for which we have more detailed clinical information. Thus, disruption of *TRPC6* might represent a predisposing genetic risk for ASD, but the penetrance of the phenotype seems to depend on additional hits, as we previously suggested [33].

In order to understand the relationship between deficiencies of *TRPC6* function due to heterozygous disruptive variants and ASD behavioral defects, we investigated the consequences on behavior of disabling *trpγ*, the *Drosophila* gene most similar to *TRPC6*. For this we focused on quantitative behaviors that are affected in ASD patients and are also abnormal in the corresponding *Drosophila* mutants, including circadian rhythms, sleep, learning and memory (reviewed [69]). We also assayed courtship behavior, as it is a complex and stereotyped behavior in which the duration of each step depends on the attention that the male pays to the female’s responses to his advances [42]. Thus, it provides a quantitative assay for selective attention, which can be altered in ASD patients [70]. We found that flies bearing null mutant alleles of *trpγ* expressed a number of behavioral defects that mirror those of *TRPC6* patients, including defective social interactions (analyzed by courtship behavior), hyperactivity (measured as locomotor activity), sleep homeostasis, and learning and memory (analyzed by courtship conditioning behavior). Importantly, most of these behavioral defects were restored by introducing a wild type copy of the *trpγ* gene, indicating that they are caused by lack of *trpγ* function.

Despite the striking behavioral differences observed in *trpγ* mutant flies vs. wild type animals, we recognize that understanding the bases of these differences will require extensive further analyses. In particular, courtship is a complex behavior that involves sensory systems (vision, olfaction, taste) and motor coordination in addition to attention [41]. In this regard, *trpγ* mutants express subtle defects in proprioception [38], which may contribute to the courtship defects observed. In addition, we found that *trpγ* mutant flies are hyperactive, which could also somehow cause the shortening of the latency to court expressed by *trpγ* mutant males. Showing that attention is specifically affected could be addressed using an independent assay, such as fixation during flight (discussed in [27]). Similarly, demonstrating that the reduced associative learning expressed by *trpγ* mutant males in a courtship conditioning assay is due to a defect in learning per se would could be addressed by examining their performance in other learning assays (see [39]).

Intriguingly, whereas the defects associated with deficiencies in *TRPC6* function occur in patients that are heterozygous for *TRPC6*, most *Drosophila* results described here were obtained using flies homozygous for *trpγ* null alleles. Furthermore, introduction of a single copy of the wild type gene into this genetic background rescued the mutant phenotype, suggesting that it behaves overall like a recessive gene. A similar situation occurs with other autosomal dominant ASD or intellectual disability conditions that have been modeled in *Drosophila* such as Phelan-McDermid syndrome; [29] see also Mariano et al. 2020 [31, 32] for cases involving other diseases. Whether the different effects of genetic perturbations observed in humans vs. model organisms simply reflects the sensitivity of assays applied to humans or reveals important differences regarding the genetic control of neural function among different species, awaits further investigation.

The behavioral defects of *trpγ* mutant flies are commonly observed in flies bearing mutations in genes that cause neurodevelopmental disorders or are homologs of genes associated with ASD [28, 31, 71–73]. Even though most of these genes, including *trpγ*, are involved in the formation, specification, and maintenance of synapses [74], the physiological and molecular mechanisms underlying these phenotypes are still poorly understood. We observed that *trpγ* deficient males displayed severe impairments in the timing of courtship behavior, although they were able to correctly execute the typical sequence of behaviors involved in courtship (e.g., following, tapping, wing vibration and licking), suggesting an altered information processing in central nervous circuits responsible for the initiation and coordination of this social behavior. This would be in agreement with the observation of impaired social behavior observed in all the *TRPC6* cases reported here. Interestingly, we found that females mutant for *trpγ* elicited less vigorous courtship from males than did normal females. This decreased attractiveness could be due to the partial masculinization of the female, consistent with “the extreme male brain theory for autism” [75, 76], a question that could be experimentally addressed in this model organism.

Given that *trpγ* is primarily expressed at the adult stage (this study) and that its human homologue shows a steady expression from pre-natal to late adulthood [77, 78], our findings suggest that the symptoms of *TRPC6* ASD patients may be due to defects in the function and not the development of the relevant neuronal circuits.

Interestingly, we observed that the severity of some behavioral defects expressed by *trpγ* mutants differed between males and females (e.g., sleep; also lifespan), revealing that *Drosophila* could be used to investigate the bases for gender differences observed in ASD symptoms that differentially affect men and women [66, 79].

The use of *Drosophila* offered an opportunity to investigate how the defects expressed by *trpγ* mutants changed with age, finding that some differences with their normal counterpart were maintained (e.g., hyperactivity), whereas others were normalized (e.g., sleep in males). Because aging involves well-conserved metabolic and physiological changes [80, 81], this information could be useful to the ASD field since there is limited information on how ASD symptoms change as patients age. Regarding the aging process, decreased expression of *TRPC6* [82] was recently reported in the blood and iPSC-derived neurons of individuals with Alzheimer disorder (AD), suggesting that *TRPC6* modulates pathways related to brain aging [82]. In ASD, some comorbidities manifest themselves during the patient’s life; for example, in Phelan-McDermid Syndrome and Fragile-X Syndrome, individuals can manifest some psychiatric symptoms during particular stages of their life, including mood and anxiety disorders, progressive loss of skills, and increased behavioral problems [83–85]. Thus, this cross-sectional study contributes to our understanding of ASD symptom progression and suggests that *trpγ* could be important in brain homeostasis throughout life.

We found that hyperforin, a TRPC6 agonist and the primary active component of the St. John’s wort antidepressant, alleviated many of the defects expressed by *trpγ* mutant flies. Interestingly, hyperforin improves learning and memory, and decreases the neurotoxicity of amyloid deposits in models of depression and AD that also show reduced *TRPC6* expression [33, 82, 86–89]. *TRPC6* is expressed throughout life in most human brain regions [77, 78], in contrast to most ASD candidate genes, which are mostly expressed during early brain development [16, 90]. This, together with our finding that *trpy* is mostly expressed in the *Drosophila* brain starting at the adult stage and that feeding flies hyperforin is effective, suggests that hyperforin may be a useful candidate therapeutic drug to test in clinical trials for the treatment of neurodevelopmental disorders associated with disruptive mutations in TRPC6. Nevertheless, the fact that most of the flies used here were homozygous for null mutant alleles of *trpy* suggests that either hyperforin is effecting its rescue by acting on a separate TRP channel or that it is acting on a different pathway. In this regard it is important to note that hyperforin is known to also inhibit the reuptake of several neurotransmitters, including serotonin, norepinephrine, dopamine, GABA, and glutamate [91, 92].

How defects in *trpγ* signaling might affect the function of the relevant neuronal circuits is currently unknown. Nevertheless, iPSC-derived neurons obtained from the index *TRPC6* patient were found to exhibit reduced Ca^2+^ influx (as would be expected for a mutation that disables a Ca^2+^-permeable cation channel such as *TRPC6*), implying that the increased courtship drive and increased locomotion we observed in *trpγ* mutant flies would be due to a reduced inhibitory input. Another consequence of disabling *trpγ* function could be a reduction in the activation of the Ras-MEK-ERK1/2 or CaMKIV pathways, which can lead to a reduction in the levels of phosphorylated CREB (cAMP response element binding protein), a transcription factor activated by *TRPC6* signaling [93–95]. In line with this hypothesis, reduced levels of phosphorylated CREB protein were seen in iPSC-derived neurons of the *TRPC6* patient here revisited [33]. Moreover, in mammalian cells, *TRPC6* silencing through ERK1/2 pathways can inhibit the translocation to the plasma membrane of Kv4.3, a voltage-gated K^+^ channel, thereby decreasing GABAergic inhibition in interneurons. Yet another possibility is that *TRPC6* dysfunction decreases the level of expression of LONP1, an enzyme involved in controlling mitochondrial fission [87]. The resulting aberrant mitochondrial elongation and deficient respiratory function would then trigger excessive reactive oxygen species (ROS) production. Most of these cellular and molecular phenotypes have been rescued by treatment with hyperforin [33, 82, 86, 87, 96]. Dysfunction of these molecular pathways is relevant for neurological conditions, including epilepsy, ASD, schizophrenia and depression [86, 87, 97, 98].

In summary, here we gathered evidence that *TRPC6* may act as a modifier gene (risk variant in an oligogenic model) for ASD and, using the fly homolog, *trpy*, showed that this gene controls behaviors throughout adult life. Furthermore, our results establish *Drosophila* as a tractable model for better understanding the etiology of ASD patients bearing mutations in the *TRPC6* gene. Our findings can now be extended by leveraging the power of *Drosophila* genetics to investigate the links between the anatomical, functional, and behavioral defects, caused by mutations in *trpy*, and by upstream and downstream targets. Indeed, much is currently known about the neuronal circuits that control *Drosophila* locomotion, courtship, sleep, learning and memory, and the aging process, so the status of these circuits can be examined in *trpy* mutants. These analyses will be especially important for disentangling the role of *trpy* in different neuronal populations. Indeed, *trpy* is expressed in a number of central (this study) as well as peripheral proprioceptive organs [38], raising the possibility that the behavioral defects observed could be due to peripheral, to central, or to combinations of central and peripheral, functions subserved by the *trpy* gene. Coupling powerful human genomics studies with higher throughput functional studies using *Drosophila* promises to facilitate the expansion of genotype and phenotype correlations in autism, as it is already doing for a growing number of diseases [99]. In addition, incorporating the use of *Drosophila* assays may be a powerful strategy to model multiple hit mutations associated with ASD.

## Supplementary information

Supplemental Information 1

Supplemental Information 2 - Statistic analysis
