# Supplementary material for: Mutations in trpγ, the homologue of TRPC6 autism candidate gene, causes autism-like behavioral deficits in Drosophila
Source: Mol Psychiatry. 2022 May 2;27(8):3328–42. doi: 10.1038/s41380-022-01555-1 (PMC9708601; doi:10.1038/s41380-022-01555-1)
Supplement: Supplementary file 1 — Supplemental Information 1 [file 41380_2022_1555_MOESM1_ESM.pdf]

SUPPLEMENTARY INFORMATION

Supplementary Table 1. Clinical re-evaluation of ASD patient bearing a mutation in *TRPC6* gene.

Patient	5 years old	17 years old
**Weight (centile)**	N/A	85 kg (p>96)
**Height (centile)**	N/A	170 cm (p>75)
**Head Circumference (centile)**	N/A	56.5 cm (p>75)
**Seizures**	no	yes
**Speech**	Few incomprehensible words - non-verbal	Few incomprehensible words - non-verbal
**ASD**	yes	yes
**ID**	yes	yes
**Learning disability**	yes	yes
**Anxiety**	no	yes
**Hyperactivity**	No	yes
**Hypotonia**	no	no
**Sleep**	Disturbed	Disturbed/ insomnia
**Gastrointestinal condition**	Severe constipation	Severe constipation
**Motor disability**	no	no
**EEG**	normal	Epileptiform activity
**MRI findings**	normal	Hippocampal asymmetry (left smaller)
**Facial dimorphism**	no	no
**Metabolic alterations**	no	no

Abbreviations: N/A: not available; ASD: autism spectrum disorder; ID: intellectual disability; EEG: Electroencephalography ; MRI: Magnetic Resonance Imaging.

A- DIOPT orthology analyses of *Homo sapiens* TRPC6

Predicted Orthologs from DIOPT v8 More on Gene2Function More on Monarch

Show only best DIOPT v8 score gene

Organism	Ortholog gene symbol	DIOPT Score	Best Score from human to model organism?	Best Score from model organism to human?	Confidence
Mouse	Trpc6 MGI IMPC PubMed	15/16	Yes	No	High
Rat	Trpc6 RGD PubMed	14/14	Yes	No	High
Zebrafish	trpc6a ZFIN PubMed	11/15	Yes	No	High
Fly	Trpgamma FlyBase PubMed	4/16	Yes	No	Moderate
Fly	trp FlyBase PubMed	4/16	Yes	No	Moderate
Fly	trpl FlyBase PubMed	4/16	Yes	No	Moderate
Worm	trp-1 WormBase PubMed	6/16	Yes	No	Moderate
Budding Yeast	YVC1 SGD PubMed	1/15	Yes	No	Low

B- Functional domains identified by DIOPT in *Homo sapiens TRPC6* and *Drosophila melanogaster trpγ*.

Gene	Domain	Region	External ID	Identity
TRPC6	trp	82..897	CDD:273311	322/868 (37%)
	ANK 1	97..126		11/32 (34%)
	ANK 2	132..161		11/29 (38%)
	ANK repeat	132..159	CDD:293786	11/27 (41%)
	ANK repeat	161..185	CDD:293786	11/27 (41%)
	ANK 3	163..189		12/29 (41%)
	ANK repeat	212..247	CDD:293786	17/35 (49%)
	ANK 4	218..247		15/29 (52%)
trpγ	trp	77..841	CDD:273311	320/848 (38%)
	ANK	113..>217	CDD:238125	47/119 (39%)
	ANK repeat	118..147	CDD:293786	11/31 (35%)
	ANK repeat	175..217	CDD:293786	21/55 (38%)
	TRP_2	227..286	CDD:285535	33/59 (56%)
	RRT14	<778..892	CDD:293680	30/114 (26%)

C- BLAST analyses of *Homo sapiens TRPC6* vs. *Drosophila melanogaster* (only the 4 most similar *Drosophila* genes are shown).

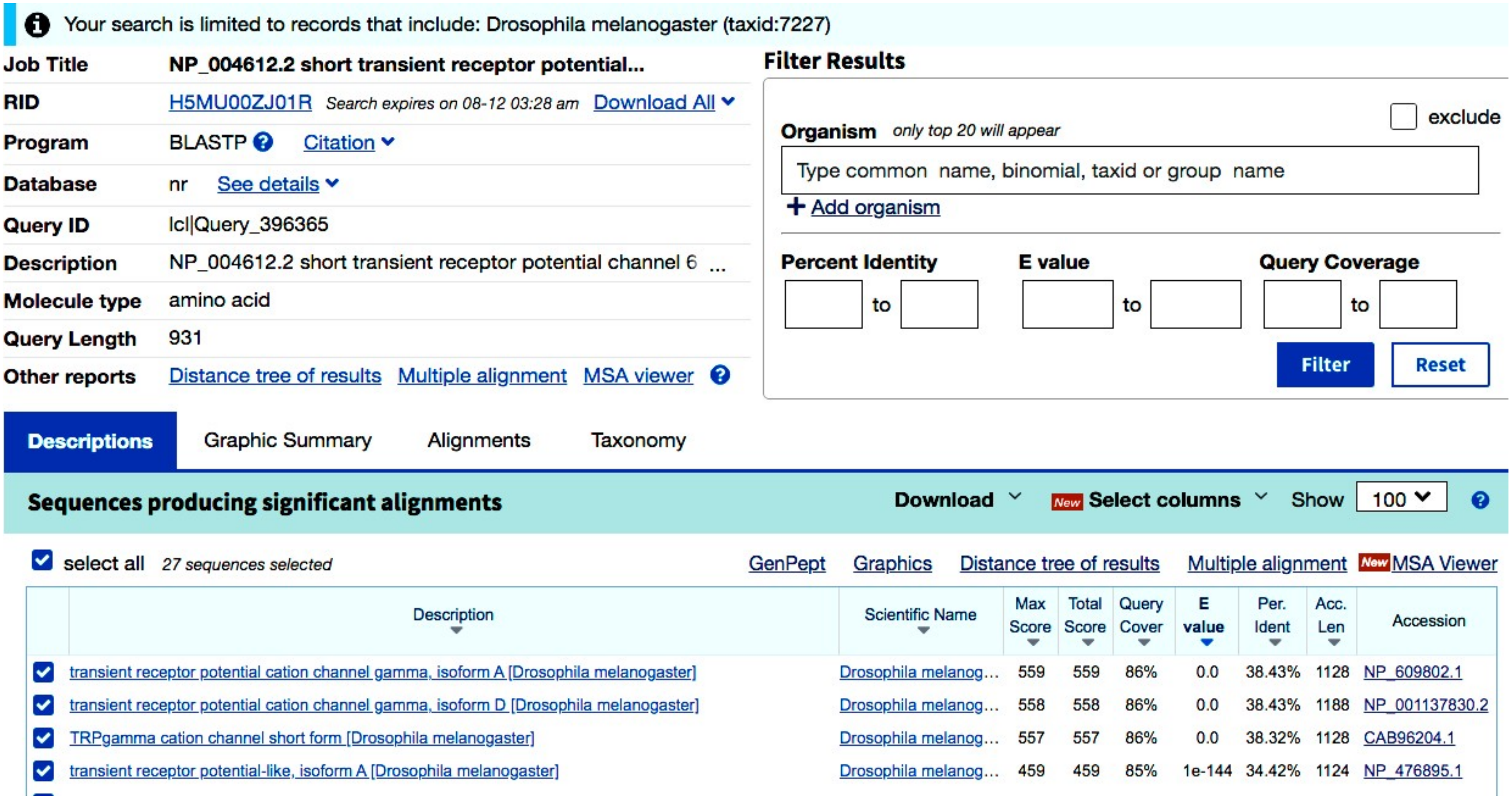

Your search is limited to records that include: Drosophila melanogaster (taxid:7227)

Job Title	NP_004612.2 short transient receptor potential...
RID	H5MU00ZJ01R *Search expires on 08-12 03:28 am* Download All
Program	BLASTP Citation
Database	nr See details
Query ID	lcl|Query_396365
Description	NP_004612.2 short transient receptor potential channel 6 ...
Molecule type	amino acid
Query Length	931
Other reports	Distance tree of results Multiple alignment MSA viewer

Filter Results

Organism *only top 20 will appear* exclude

Type common name, binomial, taxid or group name

+ Add organism

Percent Identity to E value to Query Coverage to

Filter Reset

Descriptions Graphic Summary Alignments Taxonomy

Sequences producing significant alignments Download New Select columns Show 100

select all *27 sequences selected* GenPept Graphics Distance tree of results Multiple alignment New MSA Viewer

	Description	Scientific Name	Max Score	Total Score	Query Cover	E value	Per. Ident	Acc. Len	Accession
☑	transient receptor potential cation channel gamma, isoform A [Drosophila melanogaster]	Drosophila melanog...	559	559	86%	0.0	38.43%	1128	NP_609802.1
☑	transient receptor potential cation channel gamma, isoform D [Drosophila melanogaster]	Drosophila melanog...	558	558	86%	0.0	38.43%	1188	NP_001137830.2
☑	TRPgamma cation channel short form [Drosophila melanogaster]	Drosophila melanog...	557	557	86%	0.0	38.32%	1128	CAB96204.1
☑	transient receptor potential-like, isoform A [Drosophila melanogaster]	Drosophila melanog...	459	459	85%	1e-144	34.42%	1124	NP_476895.1

D- CLUSTAL multiple sequence alignment by MUSCLE (3.8) of *Homo sapiens TRPC6 vs. Drosophila melanogaster trpγ* isoforms (abbreviated here "iso") A and D. "*" indicates identical sequences; ":" and "." indicate conservation between groups of strongly (":") or weakly (".") similar properties.

```
H_sapiens_TRPC6              MSQSPAFGPRRGSSPRGAAGAAARRNESQDYLLMDSELGEDGCPQAPLPCYGYYPCFRGS   60
D_mel_trp-gamma_iso_A        ------------------------------------------------------------   0
D_mel_trp-gamma_iso_D        ------------------------------------------------------------   0


H_sapiens_TRPC6              DNRLAHRRQTVLREKGRRLANRGPAYMFSDRSTSLSIEEERFLDAAEYGNIPVVRKMLEE   120
D_mel_trp-gamma_iso_A        ----------------------------------LTLEEKKFLLAVERGDMAGTRRMLQK   26
D_mel_trp-gamma_iso_D        ----------------------------------LTLEEKKFLLAVERGDMAGTRRMLQK   26
                                                               *::**::** *.* *::  .*:**::

H_sapiens_TRPC6              ---CHSLNVNCVDYMGQNALQLAVANEHLEITELLLKKENLSRVGDALLLAISKGYVRIV   177
D_mel_trp-gamma_iso_A        AQDTEYINVNCVDPLGRTALLMAIDNENLEMVELLINYNV   DTKDALLHSISEEFVEAV   84
D_mel_trp-gamma_iso_D        AQDTEYINVNCVDPLGRTALLMAIDNENLEMVELLINYNV   DTKDALLHSISEEFVEAV   84
                                . :****** :*:.** :*: **:**:.***:: :   . **** :**: :*. *


H_sapiens_TRPC6              EAILSHPAFAEGKRLATSPSQSELQQDDFYAYDEDGTRFSHDVTPIILAAHCQEYEIVHT   237
D_mel_trp-gamma_iso_A        EVLLDHENVTFHS---------EGNHSWESASEDTSTFTPDITPLILAAHRDNYEIIKI   134
D_mel_trp-gamma_iso_D        EVLLDHENVTFHS---------EGNHSWESASEDTSTFTPDITPLILAAHRDNYEIIKI   134
                             *.:*.*  .:  .              :..: : .** : *: *:**:***** ::***::

H_sapiens_TRPC6              LLRKGARIERPHDYFCKCNDCNQKQKHDSFSHSRSRINAYKGLASPAYLSLSSEDPVMTA   297
D_mel_trp-gamma_iso_A        LLDRGAVLPMPHDVRCGCDECVQSRQEDSLRHSRSRINAYRALASPSLIALSSKDPILTA   194
D_mel_trp-gamma_iso_D        LLDRGAVLPMPHDVRCGCDECVQSRQEDSLRHSRSRINAYRALASPSLIALSSKDPILTA   194
                             ** :** :  ***  * *::* *.::.**: *********:.****: ::***:**::**


H_sapiens_TRPC6              LELSNELAVLANIEKEFKNDYKKLSMQCKDFVVGLLDLCRNTEEVEAILNGDVETLQSGD   357
D_mel_trp-gamma_iso_A        FELSWELRRLSFLEHEFKNEYQELRKQCQDFATALLDHTRTSHELEILLNHDPTGPVYEH   254
D_mel_trp-gamma_iso_D        FELSWELRRLSFLEHEFKNEYQELRKQCQDFATALLDHTRTSHELEILLNHDPTGPVYEH   254
                             :*** **  *: :*:****:*::*  **:**...***  *.:.*:* :** *       .
```

```
H_sapiens_TRPC6             HGRPNLSRLKLAIKYEVKKFVAHPNCQQQLLSIWYENLSGLRQQTMAVKFLVVLAVAIGL   417
D_mel_trp-gamma_iso_A       GERMHLNRLKLAIKLRQKKFVAHSNVQQLLASIWYEGLPGFRRKNMALQAVDIIRIGIMF   314
D_mel_trp-gamma_iso_D       GERMHLNRLKLAIKLRQKKFVAHSNVQQLLASIWYEGLPGFRRKNMALQAVDIIRIGIMF   314
                              * :*.******* . ****** * ** * *****.* *:*::.**:: : :: :.* :

H_sapiens_TRPC6             PFLALIYWFAPCSKMGKIMRGPFMKFVAHAASFTIFLGLLVMNA--ADRFEGTKLLPNET   475
D_mel_trp-gamma_iso_A       PIFSLAYILAPYSSIGQTMRKPFIKFICHSASYFTFLFLLMLASQRIETFIGGWFFADSS   374
D_mel_trp-gamma_iso_D       PIFSLAYILAPYSSIGQTMRKPFIKFICHSASYFTFLFLLMLASQRIETFIGGWFFADSS   374
                            *:::* * :** *.:*: ** **:**:.*:**:  ** **:: :   : * *  :: :.:

H_sapiens_TRPC6             STDN-AKQLFRMKTSCFSWMEMLIISWVIGMIWAECKEIWTQGPKEYLFELWNMLDFGML   534
D_mel_trp-gamma_iso_A       GMLNTMEELPTKRGAKPTFIEWLILAWVSGLIWSEVKQLWDVGLQEYLNDMWNVIDFVTN   434
D_mel_trp-gamma_iso_D       GMLNTMEELPTKRGAKPTFIEWLILAWVSGLIWSEVKQLWDVGLQEYLNDMWNVIDFVTN   434
                            .  *  ::*   : :  :::* **::** *:**:* *::*  * :*** ::**::**

H_sapiens_TRPC6             AIFAASFIARFMAFWHASKAQSIIDANDTLKDLTKVTLGDNVKYYNLARIKWDPSDPQII   594
D_mel_trp-gamma_iso_A       SLYVATVALRVVSFFQVQKEMI-----------------YNSHATDLPRERWDAWDPMLI   477
D_mel_trp-gamma_iso_D       SLYVATVALRVVSFFQVQKEMI-----------------YNSHATDLPRERWDAWDPMLI   477
                            :::.*:.  *.::*::..*                     * :  :* * :**  ** :*

H_sapiens_TRPC6             SEGLYAIAVVLSFSRIAYILPANESFGPLQISLGRTVKDIFKFMVIFIMVFVAFMIGMFN   654
D_mel_trp-gamma_iso_A       SEGLFSAANIFSSLKLVYIFSVNPHLGPLQVSLSRMVMDIMKFFFLYVLVLFAFGSGLNQ   537
D_mel_trp-gamma_iso_D       SEGLFSAANIFSSLKLVYIFSVNPHLGPLQVSLSRMVMDIMKFFFLYVLVLFAFGSGLNQ   537
                            ****:: * ::*  ::.**: .*  :****:**.* * **:**:.::::*:.**  *: :

H_sapiens_TRPC6             LYSYYIGAKQN--------------------------EAFTTVEESFKTLFWAIFGLSEV   688
D_mel_trp-gamma_iso_A       LLWYYADLEKKRCPEVSPMSALLNMNGTNDPNACIVWRRFSNLFETTQTLFWAVFGLIDL   597
D_mel_trp-gamma_iso_D       LLWYYADLEKKRCPEVSPMSALLNMNGTNDPNACIVWRRFSNLFETTQTLFWAVFGLIDL   597
                            *  ** . :::                          . *:.: *: :*****:*** ::

H_sapiens_TRPC6             KSVVINYNHKFIENIGYVLYGVYNVTMVIVLLNMLIAMINSSFQEIEDDADVEWKFARAK   748
D_mel_trp-gamma_iso_A       DSFELDGIKIFTRFWGMLMFGTYSVINIVVLLNLLIAMMNHSYQLISERADVEWKFARSK   657
D_mel_trp-gamma_iso_D       DSFELDGIKIFTRFWGMLMFGTYSVINIVVLLNLLIAMMNHSYQLISERADVEWKFARSK   657
                            .*. ::  : * .  * :::*.*.*  ::****:****:* *:* *.: *********:*
```

```
H_sapiens_TRPC6             LWFSYFEEGRTLPVPFNLVPSPKSLFYLLLKLKKWISELFQGHKKGFQEDAEMNKINEEK   808
D_mel_trp-gamma_iso_A       LWISYFEEGGTCPPPFNIIPTPKSIWYAI----KWMRRVFCSGSSAARRE---------- 703
D_mel_trp-gamma_iso_D       LWISYFEEGGTCPPPFNIIPTPKSIWYAI----KWMRRVFCSGSSAARRE---------- 703
                            **:****** * * ***::*:***::* :    **: .:*  .  ... :.:

H_sapiens_TRPC6             KLGILGSHEDLSKLSLDKKQVGHNKQPSIRSSEDFHLNSFNNPPRQYQKIMKRLIKRYVL   868
D_mel_trp-gamma_iso_A       ---------HLKTI---------------RR-----KAQQASDRDFKYQQIMRNLVRRYVT  735
D_mel_trp-gamma_iso_D       ---------HLKTI---------------RR-----KAQQASDRDFKYQQIMRNLVRRYVT  735
                                     .*..:               *      : :. .:      :**:**:. *::***

H_sapiens_TRPC6             QAQIDKESDEVNEGELKEIKQDISSLRYELLEEKSQNTEDLAELIRELGEKLSMEPNQEE   928
D_mel_trp-gamma_iso_A       VEQRKAESQGVTEDDVNEIKQDISAFRCELVE---------------------------- 767
D_mel_trp-gamma_iso_D       VEQRKAESQGVTEDDVNEIKQDISAFRCELVE---------------------------- 767
                              * . **: * . * .:::*******::* **:*

H_sapiens_TRPC6             TNR   931
D_mel_trp-gamma_iso_A       ---   767
D_mel_trp-gamma_iso_D       ---   767
```

Supplementary Figure 1. The *Drosophila trpγ* is the fly gene most closely related to *TRPC6*. (A-B) DIOPT orthology (A) and domain analyses (B) using the sequence of the *Homo sapiens TRPC6* protein sequence. (C) BLAST analyses of *Homo sapiens* TRPC6 vs. *Drosophila melanogaster* (only the 4 most similar genes are shown). (D) CLUSTAL multiple sequence alignment of *Homo sapiens* TRPC6 and *Drosophila melanogaster trpγ* (isoforms A and D). Sequences used were: TRPC6: NP_004612.2; *trpγ* isoform A: NP_609802.1:17-783; *trpγ* isoform D: NP_001137830.2:78-844.

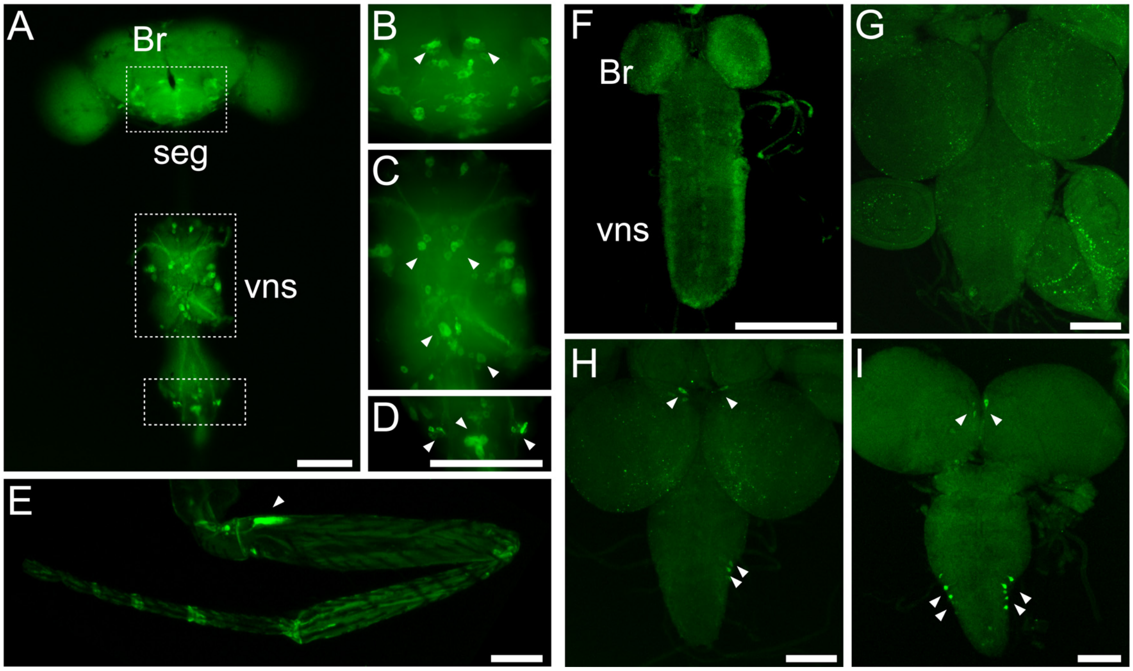

Supplementary Figure 2. *trpγ* is expressed in the adult CNS. (A-D) *trpγ* expression in the adult fly CNS; (B-D) Higher magnification views of areas of panel (A) boxed with dotted line, for the subesophageal (B), the thoracic (C), and the abdominal (D) ganglion; Arrowheads point to some of the neuronal cell bodies that express *trpγ*. (E) Expression in the adult leg; arrowhead point to *trpγ* expressing sensory neuron [41]. (F,G) Expression in second (F) and third (G) instar larval CNS; no expression was detected. (H-I) Expression in early (H) and late (I) pre-pupal CNS; arrowheads point to earliest expression. *trpγ* expression was visualized by anti-GFP immunofluorescence in *trpγ*>GFP animals; the uniform signal corresponds to autofluorescence. Scale bars: 100µm. Br: brain; seg: subesophageal ganglion; vns: ventral nervous system.

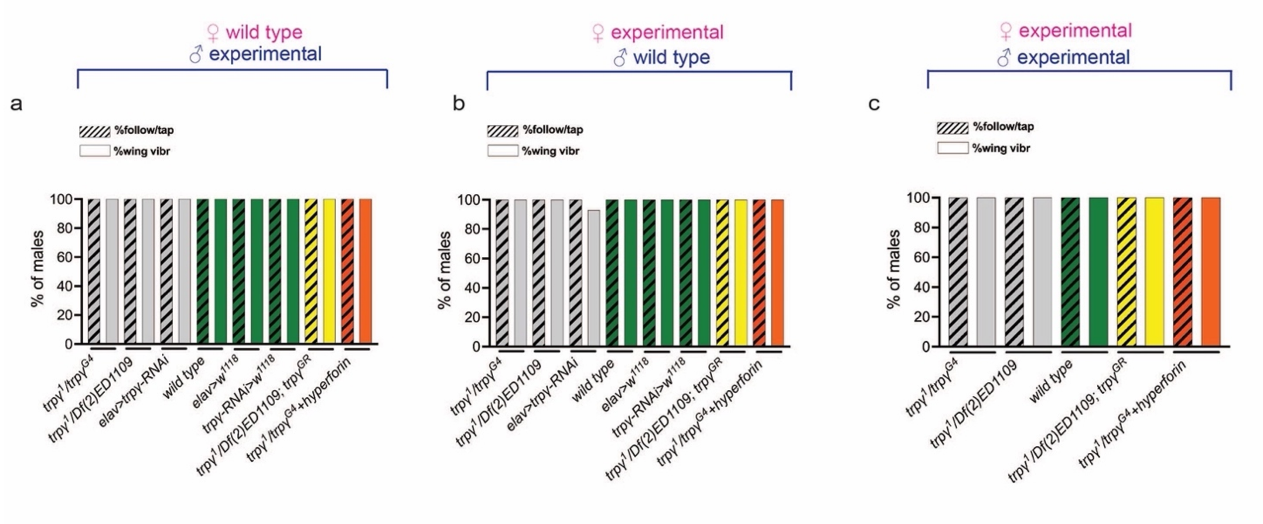

Supplementary Figure 3. Effect of mutations in *trpγ* on the following/tapping and wing vibration behaviors. Courtship ethogram (a, b, c) for pairs in which males of different genotypes were tested with wild type (*Canton-S*) females (a), pairs in which females of different genotypes were tested with wild type (*Canton-S*) males (b), and pairs in which both males and females of different (but matched) genotypes were tested (c). The percentage of males that executed following/tapping and wing vibration behaviors is indicated in striped and non-striped columns, respectively. Genotypes are indicated along X axis of panels; results are color coded such that gray columns correspond to transheterozygous null *trpγ* mutant alleles (or CNS specific trpγ knockdown), green columns correspond to relevant control genotypes, yellow columns correspond to genomic rescue of the transheterozygous null *trpγ* mutant alleles, and orange columns correspond to transheterozygous null *trpγ* mutant alleles fed hyperforin. Results are shown as means ± s.e.m.; *n* corresponds to the number of flies tested. Data were analyzed by a one-way ANOVA, followed by Tukey´s *post hoc* multiple comparison analyses where “****”: p<0.0001; “***”: p<0.001; “**”: p<0.01; “*”: p<0.05. Only the most

important statistical differences are shown in the figure. See Supplementary Table 2 for exact values for all comparisons.

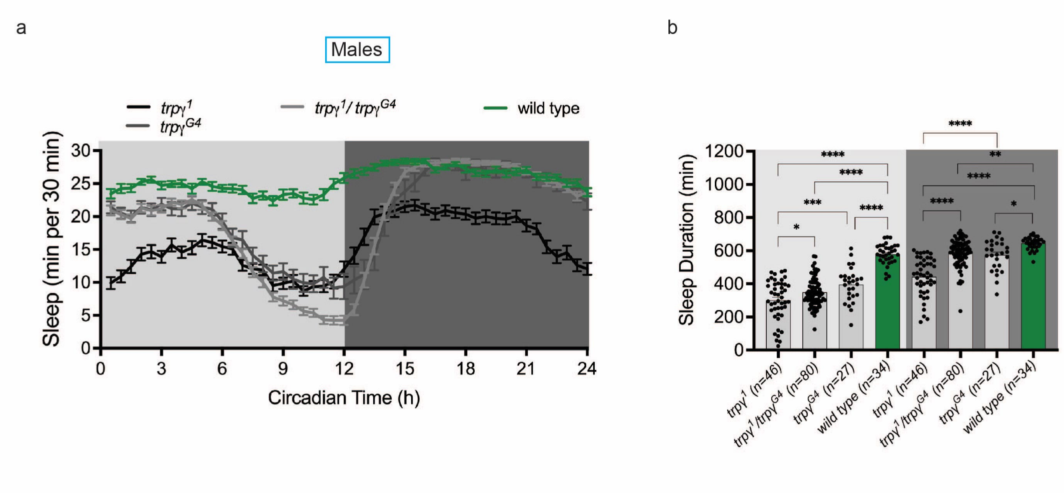

Supplementary Figure 4. Impact of mutations in *trpγ* on sleep under DD conditions in males. Average traces of sleep (a) and sleep duration (b) of males. Genotypes are indicated along the top of graph (a) and along X-axis (b); results are color coded as described in Supplementary Figure 1. Results are shown as means ± s.e.m.; *n* corresponds to the number of flies tested. Data were analyzed by a one-way ANOVA, followed by Tukey´s *post hoc* multiple comparison analyses where “****”: $p<0.0001$; “***”: $p<0.001$; “**”: $p<0.01$; “*”: $p<0.05$. Only the most important statistical differences are shown in the figure. See Supplementary Table 2 for exact values for all comparisons.

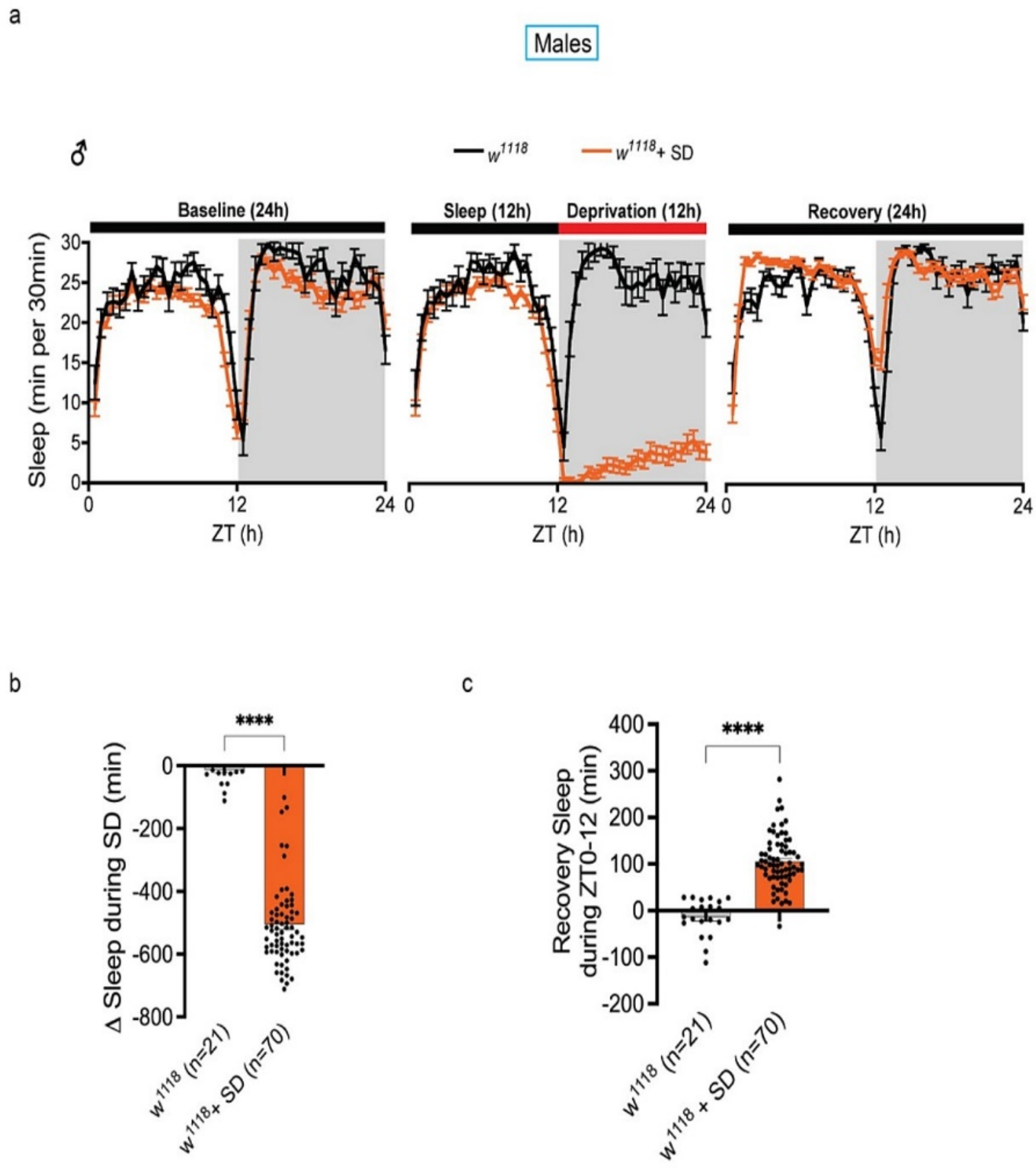

Supplementary Figure 5. Sleep, sleep deprivation (SD), and sleep recovery following SD in male *w¹¹¹⁸* control flies. (a) Average trace of sleep under LD regime (black trace) and following 12h SD (red trace). (b,c) Quantification of sleep lost during

SD (b) and sleep recovered following SD (c). Results are shown as means ± s.e.m.; *n* corresponds to the number of flies tested. Data were analyzed by two-tailed Student *t*-test, with confidence interval of 95%. See Supplementary Table 2 for exact values for all comparisons.

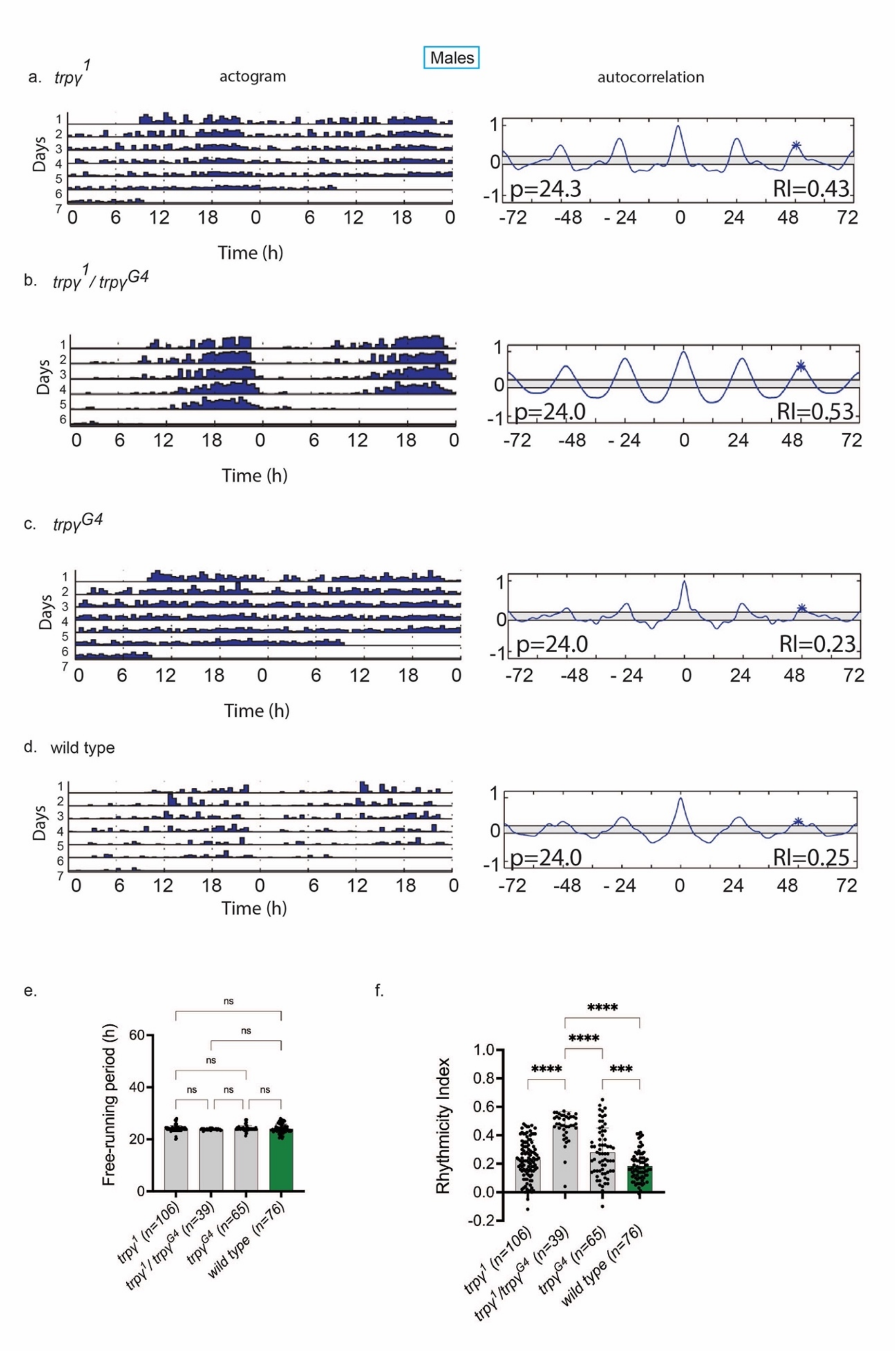
Males
a. trpγ1
actogram
autocorrelation
Days
Time (h)
p=24.3
RI=0.43
b. trpγ1 / trpγG4
p=24.0
RI=0.53
c. trpγG4
p=24.0
RI=0.23
d. wild type
p=24.0
RI=0.25
e.
Free-running period (h)
ns
trpγ1 (n=106)
trpγ1 / trpγG4 (n=39)
trpγG4 (n=65)
wild type (n=76)
f.
Rhythmicity Index
****
***
trpγ1 (n=106)
trpγ1 /trpγG4 (n=39)
trpγG4 (n=65)
wild type (n=76)

66

Supplementary Figure 6. Locomotor activity rhythms of adult male *trpγ* mutants and controls. (a-d) Examples of actograms (double plotted locomotor activity records) (left) and corresponding autocorrelogram (right; principal periodicity and rhythmicity index (RI) indicated) of adult male flies under DD conditions. (e, f) Average free-running periodicity (e) and RI (f); genotypes are indicated along X-axis and results are color coded as described in Supplementary Figure 1. Results are shown as means ± s.e.m.; *n* corresponds to the number of flies tested. Data were analyzed by a one-way ANOVA, followed by Tukey´s *post hoc* multiple comparison analyses where "****": p<0.0001; "***": p<0.001; "**": p<0.01; "*": p<0.05. Only the most important statistical differences are shown in the figure. See Supplementary Table 2 for exact values for all comparisons.

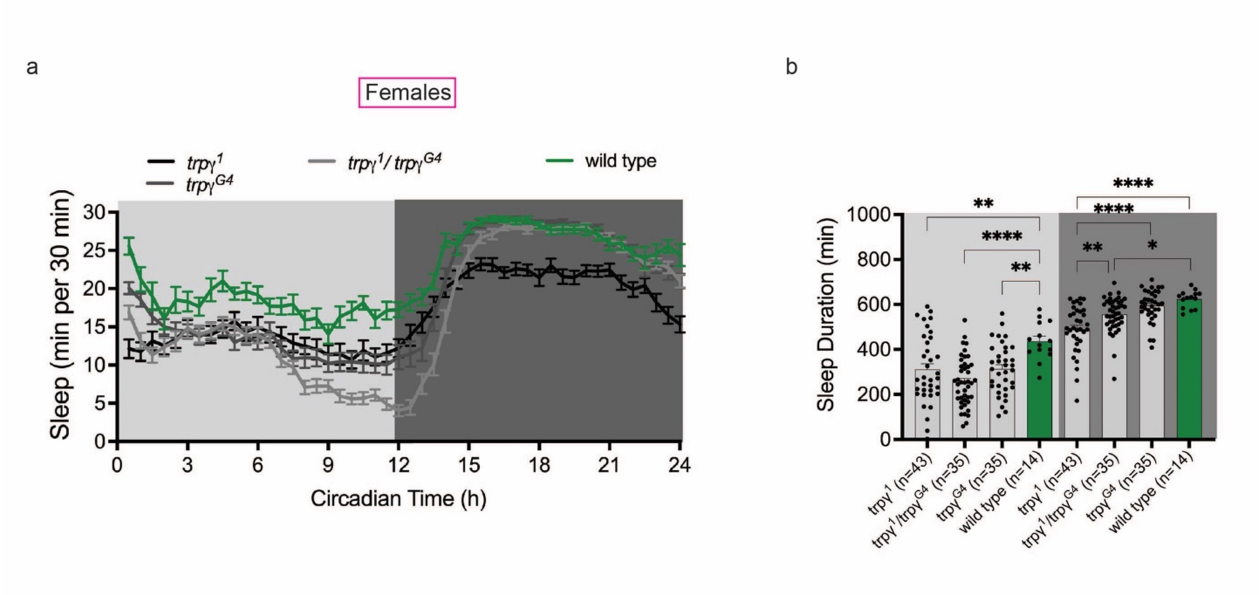

Supplementary Figure 7. Impact of mutations in *trpγ* on sleep under DD conditions in females. Average traces of sleep (a) and sleep duration (b) of females. Genotypes are indicated along the top of graph (a) and along X-axis (b); results are color coded as described in Supplementary Figure 1. Results are shown as means ± s.e.m.; *n* corresponds to the number of flies tested. Data were analyzed by a one-way ANOVA, followed by Tukey´s *post hoc* multiple comparison analyses where "****": p<0.0001; "***": p<0.001; "**": p<0.01; "*": p<0.05. Only the most important statistical differences are shown in the figure. See Supplementary Table 2 for exact values for all comparisons.

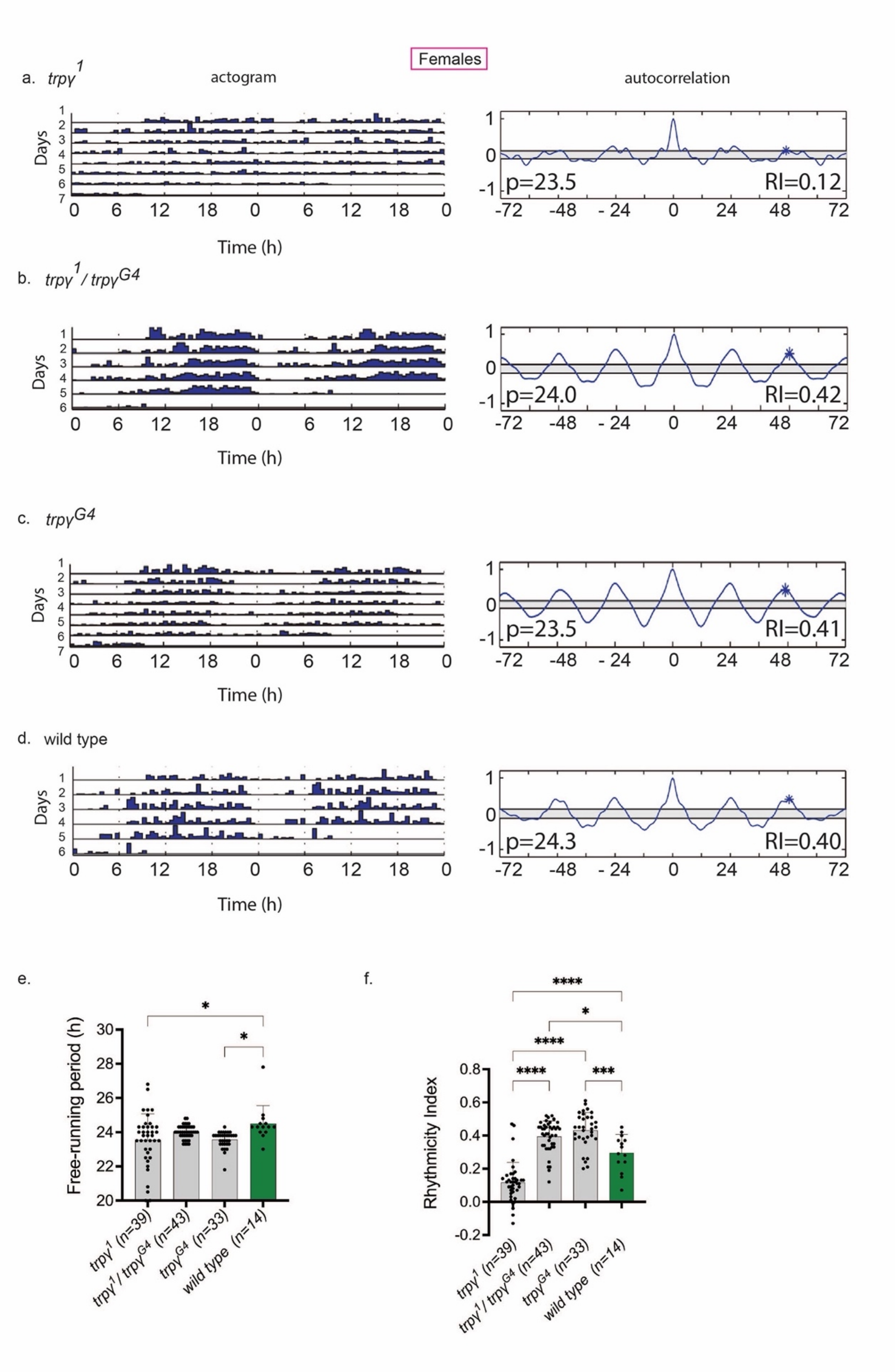

Females
a. trpγ1
actogram
autocorrelation
Days
Time (h)
p=23.5
RI=0.12
b. trpγ1 / trpγG4
p=24.0
RI=0.42
c. trpγG4
p=23.5
RI=0.41
d. wild type
p=24.3
RI=0.40
e.
Free-running period (h)
trpγ1 (n=39)
trpγ1 / trpγG4 (n=43)
trpγG4 (n=33)
wild type (n=14)
f.
Rhythmicity Index
****
***
*

Supplementary Figure 8. Locomotor activity rhythms of adult female *trpγ* mutants and controls. (a-d) Examples of actograms (double plotted locomotor activity records) (left) and corresponding autocorrelogram (right; principal periodicity and rhythmicity index (RI) indicated) of adult female flies under DD conditions. (e, f) Average free-running periodicity (e) and RI (f); genotypes are indicated along X-axis and results are color coded as described in Supplementary Figure 1. Results are shown as means ± s.e.m.; *n* corresponds to the number of flies tested. Data were analyzed by a one-way ANOVA, followed by Tukey´s *post hoc* multiple comparison analyses where "****": $p<0.0001$; "***": $p<0.001$; "**": $p<0.01$; "*": $p<0.05$. Only the most important statistical differences are shown in the figure. See Supplementary Table 2 for exact values for all comparisons.
